# Unraveling the effect of genomic structural changes in the rhesus macaque - implications for the adaptive role of inversions

**DOI:** 10.1186/1471-2164-15-530

**Published:** 2014-06-26

**Authors:** Anna Ullastres, Marta Farré, Laia Capilla, Aurora Ruiz-Herrera

**Affiliations:** Institut de Biotecnologia i Biomedicina (IBB), Universitat Autònoma de Barcelona, Campus UAB, 08193 Cerdanyola del Vallès, Barcelona Spain; Institut de Biologia Evolutiva (CSIC-Universitat Pompeu Fabra), Passeig Marítim de la Barceloneta 37-49, 08003 Barcelona, Spain; Departament de Biologia Cel·lular, Fisiologia i Immunologia, Universitat Autònoma de Barcelona, Campus UAB, 08193 Cerdanyola del Vallès, Barcelona Spain; Department of Comparative Biomedical Sciences, The Royal Veterinary College, Royal College Street, London, NW1 0TU UK

**Keywords:** Genome shuffling, Inversions, Macaque, Recombination, Adaptation, Meiosis, Tandem repeats, Evolutionary breakpoints

## Abstract

**Background:**

By reshuffling genomes, structural genomic reorganizations provide genetic variation on which natural selection can work. Understanding the mechanisms underlying this process has been a long-standing question in evolutionary biology. In this context, our purpose in this study is to characterize the genomic regions involved in structural rearrangements between human and macaque genomes and determine their influence on meiotic recombination as a way to explore the adaptive role of genome shuffling in mammalian evolution.

**Results:**

We first constructed a highly refined map of the structural rearrangements and evolutionary breakpoint regions in the human and rhesus macaque genomes based on orthologous genes and whole-genome sequence alignments. Using two different algorithms, we refined the genomic position of known rearrangements previously reported by cytogenetic approaches and described new putative micro-rearrangements (inversions and *indels*) in both genomes. A detailed analysis of the rhesus macaque genome showed that evolutionary breakpoints are in gene-rich regions, being enriched in GO terms related to immune system. We also identified defense-response genes within a chromosome inversion fixed in the macaque lineage, underlying the relevance of structural genomic changes in evolutionary and/or adaptation processes. Moreover, by combining *in silico* and experimental approaches, we studied the recombination pattern of specific chromosomes that have suffered rearrangements between human and macaque lineages.

**Conclusions:**

Our data suggest that adaptive alleles – in this case, genes involved in the immune response – might have been favored by genome rearrangements in the macaque lineage.

**Electronic supplementary material:**

The online version of this article (doi:10.1186/1471-2164-15-530) contains supplementary material, which is available to authorized users.

## Background

Large-scale genomic changes, such as inversions, translocations, fusions and fissions, contribute to the reshuffling of the genomic architecture of organisms, providing new sources of variation on which natural selection can work. In recent years, there has been an increasing number of studies focusing on the role of chromosomal reorganizations in adaptation and speciation processes [1–4], and more specifically on the influence of genome shuffling in recombination ([5] and references therein). In this framework, the “suppressed recombination” model has provided compelling evidence and a theoretical framework to explain how chromosome rearrangements are involved in speciation [6, 7]. Under this model, reorganizations such as inversions would have a minimal influence on fitness when present in the heterokaryotype, but rather would suppress recombination between genomic regions involved in reorganization, leading to the reduction of gene flow between diverging populations. In this context, chromosomal rearrangements would act as genetic barriers, interfering in the fixation of favorable alleles and allowing for the accumulation of genetic incompatibilities [8]. As a way to test this hypothesis, subsequent studies have analyzed sequence divergence (patterns of nucleotide differentiation) between species as an indirect estimation of recombination [9, 10]. High rates of sequence divergence detected in genes located at, or near, chromosomal rearrangements have often been interpreted as indirect evidence of chromosomal speciation through suppressed recombination [9–16]. However, few empirical data have focused on the relationship between evolutionary breakpoint regions (EBRs) and recombination rates. Initial studies in *Drosophila* described a strong reduction of recombination around inversion breakpoints and within the reorganization itself [17]. Analogous studies in mammals are scarce, and the role of evolutionary regions in recombination has just started to be elucidated [5, 14].

Whole-genome comparisons of distantly related mammalian species have provided the basis for establishing models that can explain genome dynamics [18–22]. In this sense, different approaches have been developed in recent years to define homologous synteny blocks (HSBs; i.e., regions where gene order has been conserved among species) and EBRs (regions where the synteny has been disrupted by chromosomal reorganizations) among mammalian genomes [19, 23–26]. Such reconstructions have revealed that genomic regions implicated in structural changes which occurred during the evolution of species are not distributed randomly through the genome, but instead they are clustered in regions that are more prone to break and reorganize [19, 23–26]. The fact that some chromosomal regions have been reused during mammalian chromosomal evolution questions (i) whether these regions are physically labile due to their DNA sequence and/or structural chromatin conformation, and (ii) whether they represent regions where selection against breakpoints is minimal [26]. Regarding the first assumption, previous studies on mammalian genomes have provided compelling evidence that EBRs can be linked to the presence of repetitive elements, such as transposable elements, segmental duplications and/or tandem repeats [19, 25–31]. However, given the diversity of repetitive elements in EBRs, it is likely that sequence composition is not alone influencing genome instability, clamoring for the involvement of additional factors such as the state of the chromatin (i.e., open chromatin may drive chromosomal reorganizations [32]) or selective constraints. In this latter case, comparative genomic studies have shown that mammalian EBRs tend to localize in gene-dense regions [22, 28, 32]. But there is a long-standing debate on the mechanisms behind this well-known phenomenon. Several lines of evidences indicate that EBRs are precisely located between genes (i.e., intergenic regions, see [32]) not necessarily affecting gene structure/function, while others have reported possible gene expression changes due to genome reshuffling (see [33]).

Given this context, the general picture of the genomic features and DNA organization of genomic regions affected by structural reorganizations is still incomplete, as is that of how genomic changes are transmitted to the offspring during the formation of germ cells and contribute to speciation. If genomic shuffling does affect evolutionary processes through the mechanical shearing at evolutionary breakpoints, how does it impact on meiotic recombination? In this sense, the analysis of the most recent human and chimpanzee recombination maps has revealed that rearranged chromosomes presented lower recombination rates than chromosomes that did not suffer any reorganization since the human-chimpanzee common ancestor [5]. Elucidating upon whether this pattern also holds for other mammalian species would have a relevant impact on our understanding of the role of genome shuffling in speciation. Here, we have analyzed the effect of genomic structural changes on genetic recombination in the rhesus macaque to understand the mechanisms underlying chromosomal evolution in mammals and determine, in the long-term, the influence of chromosomal reorganizations on meiotic recombination. To this end, we have firstly characterized the genomic regions involved in chromosomal rearrangements between human and macaque genomes. The rhesus monkey (*Macaca mulatta*, Tribe Papionini, Catarrhini) is a primate species widely used in both biomedicine and evolutionary studies [34–37]. All members of the Tribe Papionini (*Macaca*, *Papio*, *Mandrillus* and *Cercocebus*) are characterized by highly stable karyotypes that have been regarded to retain the ancestral Catarrhini karyotype [34, 35]. Due to such characteristics, the macaque has often been used as a reliable primate out-group candidate for evolutionary studies when studying great apes. But despite its importance, little effort has been made in characterizing the genomic landscape of HSBs and EBRs in this species since the initial release of the rhesus macaque genome [32, 36, 37]. Here we provide a detailed genomic map of the structural rearrangements between human and macaque. We have refined the genomic position of known rearrangements previously reported by cytogenetic approaches and described new putative micro-rearrangements (inversions and *indels*) between human and macaque genomes. Moreover, we have analyzed the repetitive DNA content and gene density in relation to chromosomal reorganizations, as well as the effect of inversions in meiotic recombination, detecting immune-related genes in evolutionary breakpoint regions in the macaque genome.

## Results and discussion

### Homologous synteny blocks (HSBs) and evolutionary breakpoint regions (EBRs) in human and rhesus macaque genomes

**Figure 1:**
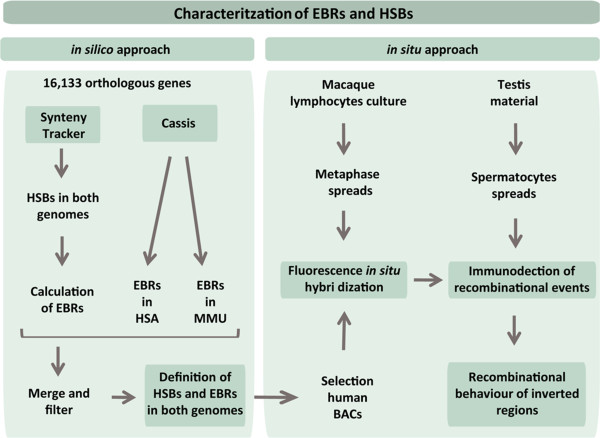
**Experimental design of the study.**

In order to analyze the chromosomal reorganizations (fusions/fissions, translocations and inversions) between human and rhesus macaque, two different algorithms were applied: *SyntenyTracker* and *Cassis* (See Material and Methods for further information). Both approaches can detect HSBs and EBRs in both species based on the positions of orthologous genes. By analyzing a total of 16,133 orthologous genes between *Homo sapiens* (HSA) and *Macaca mulatta* (MMU), *SyntenyTracker* detected 59 EBRs in both genomes, with a median length of 259 Kbp in the human genome (ranging from 11.6 Kbp to 4.6 Mbp) and 163.6 kbp in the macaque (ranging from 16.9 Kbp to 6.1 Mbp) (Table 1 and Figure 1). *Cassis*, on the other hand, detected 109 EBRs in the human genome, and 111 EBRs in the macaque (Table 1), with a median length of 51.3 Kbp for human EBRs (ranging from 3 bp to 4.5 Mbp), and 26 Kbp for the rhesus macaque (ranging from 7 bp to 512.5 Kbp) (Table 1). Merging the results obtained with both approaches resulted in 121 EBRs in the human genome and 125 EBRs in the macaque. After applying a conservative filtering process (See material and methods for further information), we obtained a total of 74 EBRs and 101 HSBs in the human genome with a median EBR length of 51.6 Kbp (ranging from 3 bp to 508.7 Kbp). Likewise, we obtained 77 EBRs and 94 HSBs in the macaque genome (Table 1 and Additional file 1: Table S1, Additional file 2: Table S2 and Additional file 3: Figure S1), with a median EBR length of 26 Kbp (ranging from 7 bp to 420.3 Kbp).

**Table 1:** **Summary of the evolutionary breakpoint regions (EBRs) detected in the human (HSA) and macaque (MMU) genomes**

	SyntenyTracker		Cassis		Merged	
	HSA	MMU	HSA	MMU	HSA	MMU
**Number of EBRs**	59	59	109	111	74	77
**Minimum length (Kbp)**	11.6	16.9	0.003	0.007	0.003	0.007
**Maximum length (Kbp)**	4,614.2	6,114.9	4,505.9	512.5	508.7	420.3
**Median length (Kbp)**	259.3	163.6	51.3	26	51.6	26

Our own study represents a departure from those conducted previously [27, 28, 30] in that it relies on a detailed comparison between human and rhesus macaque genomes based on orthologous genes and whole-genome sequence alignments. Previous cytogenetic studies delineated the primate ancestral karyotype, defining conserved syntenies among species and the direction of chromosomal rearrangements in a phylogenetic context [24, 34, 38, 39]. Species from the Tribe Papionini – including *Macaca, Papio, Mandrillus* and *Cercocebus* – are characterized by sharing the same karyotype and large-scale chromosomal reorganizations since their divergence from a common primate ancestor [34, 35]. When comparing these species with the human karyotype, previous cytogenetic studies described the presence of 20 intra- and inter-chromosomal reorganizations [34, 35, 37, 40]. These rearrangements include 12 pericentric inversions affecting eleven chromosomes, four paracentric inversions involving four chromosomes and four fusions/fissions [34, 35, 37, 40]. Overall, our *in silico* approach confirmed the presence of the above-mentioned macro-reorganizations and, thus, refined the breakpoints involved in both genomes (Additional file 1: Table S1). Moreover, we identified 39 and 41 previously undetected EBRs in the human and rhesus genome, respectively, affecting 13 different chromosomes (Additional file 1: Table S1). This resulted in 21 previously undetected reorganizations in the human genome and 23 in the macaque. Although previous cytogenetic studies have reported that six chromosomes (MMU6, MMU8, MMU11, MMU17 and MMU19) have been maintained collinear between both species, our results suggest that only chromosomes MMU6, MMU12 and MMU17 have maintained a complete conserved synteny. Six new *indels* (insertions or deletions) were also identified, ranging from 3.3 Kbp to 2,784.8 Kbp in five different chromosomes (Additional file 1: Table S1).

### Tandem repeats do not accumulate in EBRs in the macaque genome; instead, they are correlated with the evolutionary history of chromosomes

Previous comparative genomic studies have revealed that mammalian breakpoint regions are especially rich in repetitive elements, such as segmental duplications [41–43], repetitive sequences [25, 42], transposable elements and long regulatory regions [26, 29, 44–46]. We tested whether this pattern applies to the rhesus macaque, a species that has retained the ancestral Catarrhini karyotype [34, 35]. To this end, the genome distribution of tandem repeats (TR) in this species was analyzed. A total of 701,128 *loci*, representing 60.9 Mbp of the whole genome was detected. In order to study the genome-wide distribution of TR, the number of base pairs involved in TR by screening non-overlapping windows of 100 Kbp along the genome was analyzed. When considering the number of base-pairs (per 100 Kbp) of each window covered by TR, we observed a significant increase of TR in telomeric and centromeric regions when compared to HSBs and EBRs (Kruskal-Wallis test, p-value < 0.0001), mirroring previous observations obtained in the human and great apes [25, 26]. Subsequently, we tested whether there was a correspondence between TR and EBRs in all macaque chromosomes. Our analysis indicated that EBRs are not significantly enriched by tandem repeats when compared to HSBs (Mann–Whitney U test, p-value > 0.05).

Recent studies in great apes have found differences in the genome-wide distribution of TR among species, suggesting that they might be correlated with the evolutionary history of each primate chromosome [26]. More specifically, qualitative comparisons of TR distribution in great apes indicated that the TR landscape might have been conserved in collinear chromosomes, but altered in those reorganized chromosomes [26]. Under this assumption, genomic regions that have suffered more rearrangements during their evolution are expected to concentrate more repetitive sequences than are conserved regions. In fact, our dataset supports this assumption since rearranged chromosomes in the macaque lineage (MMU3 and MMU5) have more TRs than do those that maintained the ancestral form (MMU2, MMU7, MMU10, MMU12, MMU13 and MMU18) (Mann–Whitney U test, p-value < 0.0001). Despite the limitations of the current rhesus macaque draft genome assembly and annotation [47, 48], this view is consistent with the lack of differences found in TR density between EBRs and HSBs in the macaque, which has maintained an ancestral karyotype within Catarrhini and, consequently, can be considered to have retained a more conserved chromosome complement (i.e., low degree of genome reshuffling) than have those of great apes [25, 26].

### Defense-responsive genes are over-represented in EBRs

Once the evolutionary genomic landscape of the macaque was established, the genome-wide distribution of genes was further examined, paying special attention to gene ontology. A total of 28,595 genes was included in the analysis: 21,023 protein-coding genes; 5,913 non-coding RNA genes and 1,659 pseudogenes. We scrutinized each macaque chromosome’s complete sequence using non-overlapping windows of 100 Kbp in order to analyze the distribution of genes genome-wide. The mean distribution is 0.98 genes per 100 Kbp (including protein-coding genes, non-coding RNA genes and pseudogenes) in the whole macaque genome. When analyzing the distribution of protein-coding genes in more detail, a higher gene density in EBRs was detected (1.48 genes/100 Kbp), when compared with HSBs (0.73 genes/100 Kbp) (Kruskal-Wallis test, p-value < 0.0001). Therefore, our results indicate the presence of EBRs in gene-rich regions, in line with previous observations in mammals using multi-species comparative maps [22, 32]. In trying to understand the reasons behind this pattern, initial studies reported the intergenic location of mammalian EBRs [22], while recent studies have paid special attention to the adaptive role of EBRs [33]. This has been the case of the pig, for example, where EBRs have been found to be especially rich in taste perception networks [49], suggesting that genome reshuffling significantly contributed to adaptation and the development of lineage-specific traits. Moreover, it has also been suggested that inversions can suppress recombination within the affected zones [5–8]. Consequently, rearranged sequences could accumulate alleles, which might be adaptive for the population, and this could generate reproductive isolation leading, eventually, to speciation.

With this in mind, the function of the genes located at or near EBRs was analyzed in the macaque genome through the analysis of GO terms enrichment. We focused our study on the genes located in the positions of the EBRs detected *in silico*, considering flanking regions of ±200 Kbp in size in order to cover genes that overlapped EBR starts and/or ends. We applied the Functional Annotation Clustering Tool from DAVID database [50] using a background list containing 19,794 protein-coding genes. This was created by filtering the total protein-coding genes list available in the database, discarding 2,111 genes located in the centromeres and telomeres. Using this approach we found that macaque EBRs are enriched in genes related to the immune system. In fact, we detected a single functional module significantly associated to EBRs (Enrichment Score = 2.81, Table 2 and Additional file 4: Table S3). This functional module included 17 genes implicated in the immune response (including several GO terms, such as chemokine, defensin precursors and Toll-like receptor signalling pathway, among others) located in seven different macaque EBRs (Table 2, Additional file 4: Table S3). Interestingly, five of these genes are beta-defensins, and are clustered in MMU10 EBR involved in the pericentric inversion that occurred after macaque and human divergence (Table 2). Beta-defensins are antimicrobial peptides involved in the resistance to microbial colonization of the epithelial surface [51]. This cluster, whose expression is restricted to the male reproductive tract, is the result of a series of duplication events subsequently shaped by the action of positive selection [52, 53]. In fact, previous studies have shown a marked differential expression of DEFB118 and DEFB122 (two of the genes detected in MMU10, Table 2) in human and macaque reproductive tissues [50]. Since chromosomal rearrangements are sources of genome variation, it might be possible that they could have influenced the structure of regulatory regions in those genes near EBRs. Moreover, and in line with these observations, we also identified an alfa-defensin cluster in the vicinities of the EBR detected in MMU8 (Table 2). This gene cluster has been previously described as an example of rapid evolution in primates [54]. Developing protection to new microbial infections is among one of the great challenges that species confront during the adaptation to new ecological niches. Thus, the generation of new defensin variants through different mechanisms, such as sequence modifications when a chromosomal reorganization occurs, could lead to adaptation to new environments. In fact, defensin clusters have also been described as being located in EBRs in Cetartiodactyla [55], suggesting the importance of genome reshuffling as an important source of new gene variants.

**Table 2:** **Genes located in macaque EBRs**

Gene	Distance from EBR edges (Kbp)	EBR position (chr: start-end)	Function
GNRHR2	147.5	MMU1: 126,278,886-126,466,180*	Gonadotropin receptor
ADIPOQ	156.7	MMU2: 179,026,672-179,049,651*	Adiponectin precursor
F7D492	133.6	MMU8: 8,165,750-8,338,584	Defensin precursors
DEFA4	164.6		
LOC574310	172.8		
MSMB	31.1	MMU9: 46,613,935-46,645,068	Beta-microsemino protein
DEFB123	189.6	MMU10: 33,312,300-33,313,023*	Beta-defensins
DEFB118	122.5		
DEFB119	113.7		
DEFB121	152.7		
DEFB122	171.5		
CCL18	129.4	MMU16: 31,482,483-31,663,199*	C-C motif chemokine precursors
CCL23	193.7		
CCL3	106.4		
Q76LL8	182.8		
CCL25	106.1	MMU19: 7,76,0383-7,770,483	C-C motif chemokine precursor
DC-SIGN	53		Pathogen-recognition receptor

Protein-coding genes of the single functional annotation term (Enrichment Score = 2.81) detected in the proximity of EBRs in the macaque genome. *macro-reorganizations; MMU – macaque chromosome.

Moreover, we also investigated genes located within the inverted regions that are specific for the macaque linage, as they could have played an adaptive role in this species. Statistically significant enrichment was detected (Enrichment Score ≥ 1.5) for defense-response genes within the paracentric inversion affecting MMU5, a chromosomal reorganization that has been fixed in the lineage leading to macaque (Table 3 and Additional file 5: Table S4). MMU5 inversion spans approximately 33 Mbp across the centromere, involving one breakpoint located between 44.38-44.44 Mbp and the other one located between 77.5-77.9 Mbp (Table 3). Specifically, we identified two different statistically significant functional modules consisting of 13 and 6 genes, respectively – mostly coding for chemokines and the UDP-Glucuronosyltransferases 2B gene family –out of a total of 181 protein-coding genes present within the rearranged region (Enrichment scores: 4.72 and 3.52, respectively) (Table 3 and Additional file 5: Table S4). Both functional modules detected include immune-response genes that might have been influenced by the chromosomal rearrangement in the rhesus monkey. Chemokines are known to play a role in the neuroinflammation process in response to infection, are present in the central nervous system (CNS) and are expressed in neurons and glial cells [56]. Moreover, there is evidence suggesting that they are also involved in neurodevelopment and neurophysiological signaling [56, 57]. UDP-Glucuronosyltransferases, on the other hand, are enzymes from the major pathway for the elimination of xenobiotics and endobiotics, and it has been suggested to play a role during speciation in the lineage leading to macaque [58].

**Table 3:** **Genes located in the MMU5 inversion**

Gene	Distance from EBR	EBR position (start-end)
**Term 1 (ER = 4.72)**		
CXCL13	7,573.4	44,386,928-44,442,153
CXCL11	9,135.6	
CXCL10	9,176.8	
CXCL9	9,193.0	
CXCL3	11,230.2	
PPBP	11,276.2	
PF4	11,282.6	
CXCL1	11,420	
PF4V1	11,439.3	
P67813	11,555.7	
ALB	11,867.3	
ODAM	15,090.7	
KIT	2,815.7	77,560,198-77,980,505
**Term 2 (ER = 3.52)**		
SULT1E1	15,330.9	44,386,928-44,442,153
UGT2B4	17,318.6	77,560,198-77,980,505
UGT2A3	17,222.2	
UGT2B33	17,078.8	
UGT2B15	16,769.3	
SRD5A3	3,533.6	

Protein-coding genes contained in the two GO terms detected in the macaque-specific pericentric inversion affecting chromosome MMU5. ER = Enrichment Score.

Our observation of an over-representation of defense-responsive genes in both EBRs and macaque-specific inversions might suggest an adaptive role of reorganizations in this species. Previous studies have reported that a small proportion of the mammalian genome, i.e., 4% in the case of the human genome, is under selective constraints, especially so for coding regions, introns and intergenic regions [59]. This suggests that in certain regions the fitness cost is so pronounced (i.e., could be lethal or deleterious for the individual and the progeny) that rearrangements are not allowed (i.e., [60]). But it has also been shown that this constraint could be somewhat relaxed in the promoters of genes linked to the immune system, reproduction and perception [59], allowing for the generation of new variability to ensure adaptation to new environments. In light of our results, this might be the case for the genomic regions under study in the macaque. However, whether immune-related genes are directly involved in lineage-specific adaptation, as has been previously suggested for macaque [58], needs further validation.

### Genome reshuffling and its effect on chromosome-specific recombination landscapes

Once the genomic structural changes were defined in the rhesus macaque, together with the genome distribution of coding-genes across evolutionary regions, we further experimentally analyzed the meiotic recombination landscape as a way to explore the adaptive role of chromosomal changes. Under the “suppressed recombination” model of chromosomal evolution, chromosome rearrangements would have a minimal influence on fitness, but would rather suppress recombination within the genomic regions affected, thus contributing to the accumulation of gene incompatibilities [2, 5–9]. The analysis of the most recent human and chimpanzee recombination maps inferred from genome-wide single-nucleotide polymorphism (SNP) data revealed that the standardized recombination rate was significantly lower in rearranged rather than in collinear chromosomes [5]. In the case of rhesus macaque, chromosome-specific recombination maps are available for very few chromosomes [61], and whether or not chromosomal reorganizations that have been fixed in the macaque lineage have affected the recombination landscape was addressed in our study.

**Figure 2:**
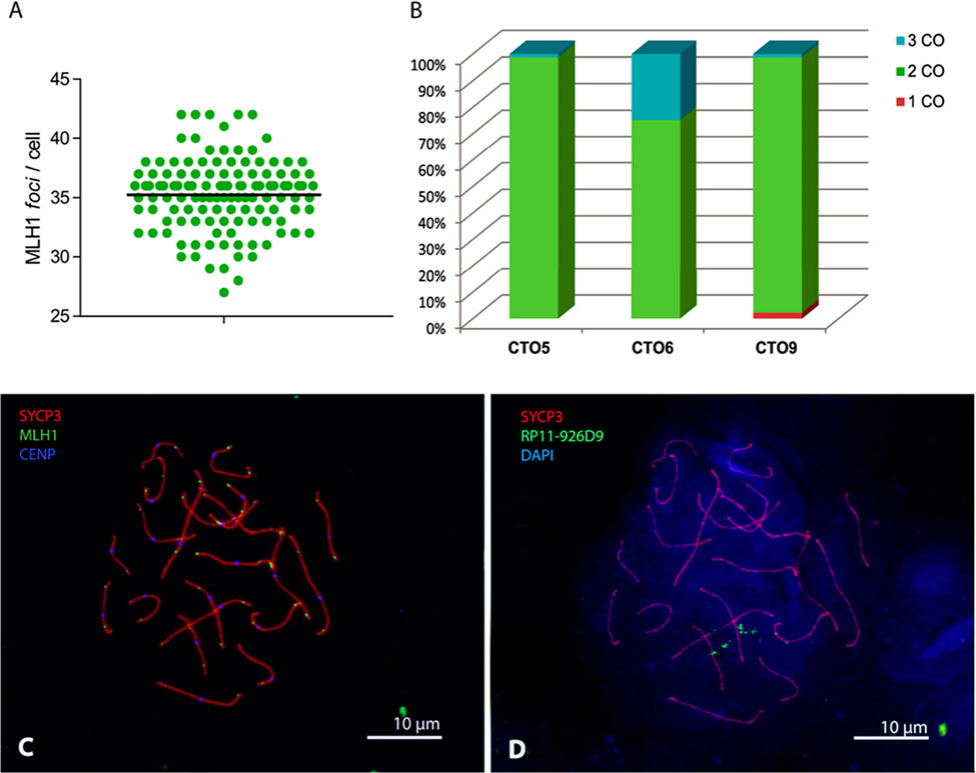
**Recombination features in** ***Cercocebus torquatus*** **. (A)** Number of MLH1 *foci* detected per cell in *Cercocebus torquatus* (CTO). The horizontal bar indicates the mean. **(B)** Comparison of the percentage of cells with a different number of MLH1 *foci* (crossovers, CO) in the different chromosomes analyzed: CTO5, CTO6 and CTO9. **(C-D)** Sequential image of a spermatocyte at pachynema from *Cercocebus torquatus* depicting **(C)** triple immunostaining against SYCP3 (red), MLH1 (green) and centromeres (blue). **(D)** Representation of the same cell after applying sequential fluorescent *in situ* hybridization (FISH) with the BAC probe RP11-926D9, specific for CTO9.

Catarrhini monkeys, and more specifically the Tribe Papionini, are characterized by the presence of highly conserved karyotypes in terms of diploid number and chromosome homologies [62, 63]. It has been described that species sharing the same karyotype (i.e., no major genome reshuffling) maintain the chromosomal distribution of meiotic crossover in conserved chromosomes on a broad scale (Mbp, the resolution provided by the *in situ* immunoflourescence detection of meiotic proteins) [64, 65]. Therefore, we expect that the recombination pattern in *Macaca* and *Cercocebus* chromosomes (species that share the same karyotype and belong to the Tribe Papionini [62, 63]) are conserved at the Mbp resolution, thus allowing us to extrapolate the results obtained in both species, as previously described [64, 65]. In this context, and taking advantage of the EBRs detected *in silico* in the macaque genome, we subsequently studied the chromosomal distribution of meiotic crossover (COs – here exemplified as MLH1 *foci*) in macaque chromosomes affected by inversions since their Catarrihini common ancestor (MMU5). This was done by combining the immunostaining of meiotic proteins and fluorescence *in situ* hybridization (FISH) with specific BAC clones spanning the EBRs detected in our *in silico* scanning (Figure 2, Table 4 and Additional file 6: Table S5). The analysis of COs distribution according to centromere positions and EBR-specific BAC probes allowed us to experimentally determine the chromosome position of evolutionarily reorganized regions directly on pachytene chromosomes along chromosomal axes. Our efforts were concentrated on three chromosomes, which represented three different evolutionary states: (i) a chromosome with a pericentric inversion (CTO5/MMU5) specific for the *Cercocebus* and *Macaca* lineages; (ii) a paracentric inversion (CTO9/MMU9) that has been fixed in human and chimpanzee lineages, and therefore has been maintained collinear in the macaque lineage; and (iii) a collinear chromosome (CTO6/MMU6) that has been maintained collinear since the Catarrhini common ancestor. With this in mind, our main goal was two-fold: (i) reconstruct chromosome-specific recombination maps, and (ii) encompass the distribution of meiotic crossovers across the inverted regions in order to test whether chromosomal inversions have an effect in reducing recombination.

**Table 4:** **Chromosome-specific recombination analysis**

Chr	N	μm SC		MLH1 ***foci***/SC	MLH1 ***foci***/μm	MLH1 ***foci/***μm	
		p-arm	q-arm			Inside inv	Outside inv
CTO5	87	3.85	4.01	2.01	0.26	0.04	0.31
CTO6p	85	2.26	6.12	2.25	0.27	0.06	0.30
CTO6q	85	2.26	6.12	2.25	0.27	0.09^#^	0.36
CTO9	86	3.19	5.56	1.99	0.23	0.03*	0.31

Chr: Chromosome, CTO: *Cercocebus torquatus*, N: number of cells analyzed, SC: synaptonemal complex, Inv: inverted region. CTO6p: inversion simulated in the small chromosome arm. CTO6q: inversion simulated in the long chromosome arm. # CTO6 density (expressed as MLH1 *foci /*μm) inside and outside the inversion refers to the simulated region for the comparison in each case. *indicates statistical significance (p-value < 0.05) when comparing reorganized region with simulated inversion in the collinear chromosome CTO6 (Mann Whitney U test).

**Figure 3:**
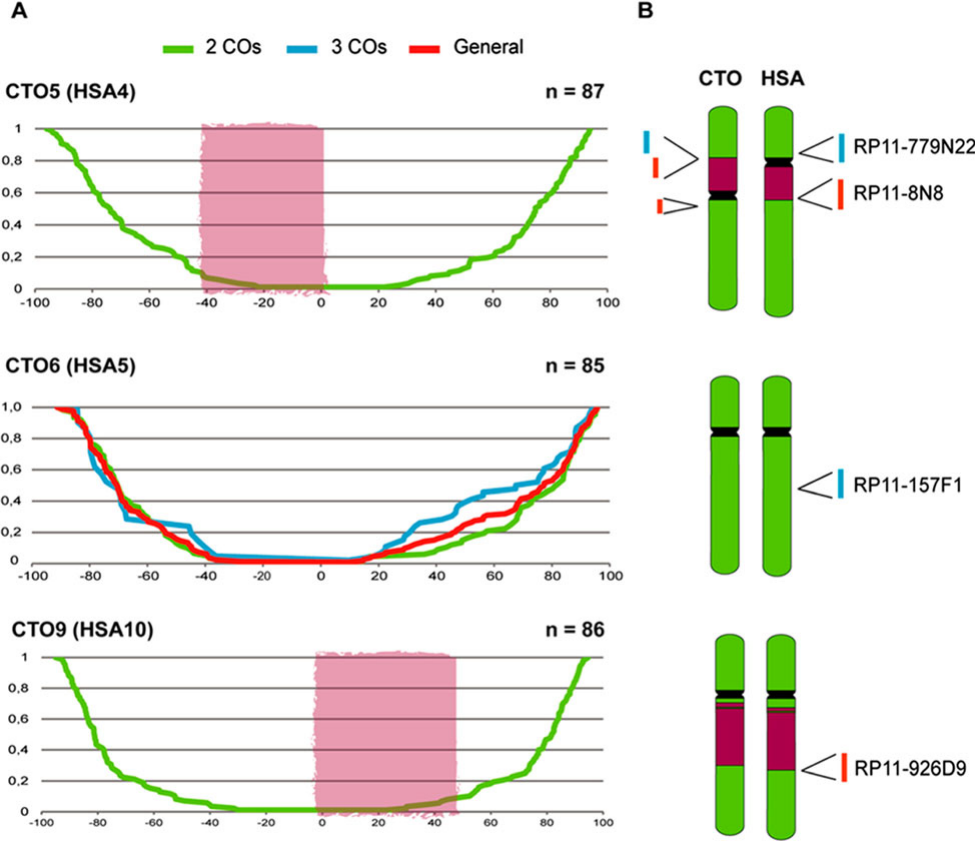
**Chromosome-specific recombination maps. (A)** Cumulative frequency graphs representing the observed meiotic COs for each chromosome analyzed (CTO5, CTO6 and CTO9). X-axis represents SC length (in%), position 0 refers to centromeres. Y-axis indicates the frequency of the COs for each position in the SC. The chromosomal regions involved in the rearrangement are depicted as red shadowed areas. Green lines represent the cumulative frequency distribution when two MLH1 *foci* (COs) are present along the SC, whereas the blue line corresponds to three COs. Red lines depicts the cumulative frequency distribution of all COs detected in CTO6. **(B)** Schematic representation of chromosomes analyzed, depicting collinear regions in green and inverted regions in purple. BACs used for the chromosome identification are depicted in each case for CTO (left) and HSA (right): RP11-779 N22 and RP11-8 N8 for CTO5, RP11-157 F1 for CTO6 and RP11-926D9 for CTO9.

A total of 258 spermatocytes at pachynema was analyzed in order to obtain chromosome-specific recombination maps. We detected an average of 35.24 (±3.01) MLH1 *foci* per analyzed cell (Figure 2). Assuming that one MLH1 *focus* can be translated into 50 centimorgans (cM) in genetic length according to previous studies [61, 64–66], these recombination events correspond to an autosomal genetic length of 1,760 cM for *C. torquatus*, very similar to the 1,950 cM obtained in *M. mulatta* [61]. Our results also indicated that all chromosome pairs present, at least, one recombination event, with an average number of 1.8 COs per homologous autosomic chromosome and 0.88 COs per autosomic arm. Moreover, a maximum of three recombination events in the large chromosomes was detected (Figure 2). In addition, for each chromosome analyzed, its total length (expressed in μm) and the density of COs (MLH1 *foci*/μm) per chromosome and chromosomal arm were calculated (Table 4). All three chromosomes presented equivalent CO densities along their synaptonemal complexes: 0.26 COs/μm for CTO5, 0.27 COs/μm for CTO6 and 0.23 COs/μm for CTO9. In general, we detected an increase in CO frequency towards the chromosomal telomeric regions, whereas centromere regions showed very low CO frequencies (Figure 3), mirroring previous studies in mammalian species [61, 64–66].

Subsequently, CO density was analyzed inside and outside each inverted region using the selected BAC probes to label the location of the breakpoint in each chromosomal region affected by the inversion (Figures 2 and 3). As a general trend, we observed low CO densities within inverted regions (0.04 and 0.03 MLH1 *foci*/μm for CTO5 and CTO9, respectively), when compared to chromosomal regions outside the reorganized area (0.31 COs MLH1 *foci* per μm in both cases) in both rearranged chromosomes (Table 4). When analyzing the recombination rate within inverted regions among chromosomes, significant reduction of CO density was observed in CTO5 and CTO9 within the inverted regions, when compared with regions outside the inversion (Table 4, Mann–Whitney U test, p-value < 0.05). Such differences were not observed in the simulated inversion in CTO6, a chromosome that has been maintained collinear in the macaque lineage (Table 4).

Subsequently, we tested whether the suppression of recombination observed within reorganized areas was due to the low recombination rate characteristic of pericentromeric regions. To do so, we considered as a pericentromeric region an area extending 30% of each chromosome arm from the centromere towards the telomeric region and compared it to the CO density observed in the same region in the collinear chromosome (CTO6). When all chromosomes were compared, our results showed no statistical differences among the pericentromeric regions in the small arms (CTO5p, CTO6p and CTO9p) (Kruskal-Wallis test, p-value > 0.05); however, differences in CO density were significant when considering the long arms (CTO5q, CTO6q and CTO9q) (Kruskal-Wallis test, p-value = 0.018). Moreover, a significant reduction was observed in the CO density within the rearranged region in CTO9, when compared to the collinear chromosome CTO6 (Mann–Whitney U test, p-value = 0.016), (Figure 3 and Table 4). These differences were not significant, however, when the inverted region of CTO5 was compared to the collinear chromosome CTO6 (Mann–Whitney U test, p-value > 0.05).

What are the evolutionary implications of our observations in light of the “recombination suppression” model? Despite the fact that the “recombination suppression” was initially proposed to explain differences in recombination rates within reorganized genomics in heterokaryotypes (i.e., heterozygotes), we observed a reduction of recombination in fixed rearrangements, raising intriguing questions about the mechanisms involved. Previous studies in great apes have revealed that rearranged chromosomes presented significantly lower recombination rates than do chromosomes that have been maintained collinear since a common ancestor, and this was related to the lineage in which they become fixed [5]. Importantly, inverted regions had lower recombination rates than did collinear and non-inverted regions, independently of the effect of centromeres [5]. Although at this stage it would be premature to argue about the evolutionary forces behind this pattern, our results highlight the importance of the study of recombination framed by the evolutionary history of chromosomes and, in greater extent, genomes. Incorporating more chromosomes into the experimental study would be necessary to detect a clearer genomic effect of the inversions in the distribution of recombination patterns.

## Conclusions

Genomic rearrangements might play an important role in local adaptation and species divergence by the modification of both the structure and regulation of genes located near the affected regions. Here, we provide a highly refined description of the chromosomal reorganizations and evolutionary breakpoint regions in the human and rhesus macaque genomes based on orthologous genes and genome sequence alignments. The high-resolution map of EBRs and HSBs defined in the macaque genome has revealed an interesting pattern: evolutionary breakpoints are gene-rich regions, with a significant functional clustering for genes related to the immune system. Furthermore, and in light of our observations, inversions can induce a reduction in the recombination rate among the different alleles contained in the inversion, which could be favoring adaptive alleles. Future comparative research on the effect of chromosomal reorganization on recombination as outlined above should be an effective means to enhance our knowledge of the role of genome reshuffling in evolution.

## Methods

### Whole-genome comparisons and evolutionary breakpoint definition

The experimental design of the study is represented in Figure 1. In order to detect the evolutionary breakpoint regions (EBRs) and homologous synteny blocks (HSBs) between human and macaque whole-genome sequences, two different algorithms were applied: *SyntenyTracker* [67] and *Cassis* [68]. Both approaches compare the order and orientation of orthologous markers (genes) between genomes, detecting changes both in the sequence order and the position of the HSBs and EBRs. Orthologous genes between human (GRch37.p7) and rhesus monkey (MMUL_1.0) genomes were obtained through the BioMart database (http://www.ensembl.org/index.html). *SyntenyTracker* [67] determines the position in the chromosome sequences of both genomes, providing information of the relative orientation of each HSB. This enables the detection of chromosomal rearrangements such as inversions and/or translocations between two genomes. Once the HSBs were detected, genomic regions between consecutive HSBs were considered EBRs. *Cassis* [68], on the other hand, is especially designed to define breakpoint regions, providing information about different types of rearrangements, such as inversions, translocations or *indels*. Both algorithms were applied as previously described [5] using default parameters for *SyntenyTracker* and level 1 in the *lastz* alignment in *Cassis*. In order to obtain the genomic positions of EBRs in both genomes, the analysis was performed in two directions: (i) using both the human genome as reference genome, and (ii) the macaque genome as reference. Following previous studies [19, 22], we considered EBRs that were 4 Mbp in size or less. Regions larger than 4 Mbp in size were considered “gaps”. Furthermore, we labeled as telomeric/subtelomeric the 2 Mbp at the ends of each human chromosome and as centromeric/pericentromeric the 2 Mbp regions flanking the unknown nucleotides (Ns), as previously described [25].

Once the genomic positions of EBRs were obtained, we followed conservative criteria in order to avoid false positives. To do so, EBRs located at telomeres, centromeres and gaps were excluded from the analysis. The resulting EBRs were classified according to whether they are involved in macro-rearrangements (rearranged regions > 1.4 Mbp) or micro-rearrangements (rearranged regions < 1.4 Mbp). Simultaneously, we also classified each EBR depending on which type of chromosomal rearrangement was involved, that is, inversion, fusion or fission, following previous studies [30].

### Analysis of repetitive elements and gene screening

The distribution of TR in the macaque genome using the eTandem algorithm was analyzed (part of EMBOSS 6.0.1 [69]). The eTandem algorithm was run with a minimum repeat unit of 2 bp and a maximum repeat unit of 100 bp, as previously described [26]. The resulting output files were computed for the detection of overlapping TR, and the canonical motif was defined using in-home Perl scripts. In order to compare the distribution of TR along macaque chromosomes, we counted the base pairs of tandem repeats in 100 Kbp windows for each chromosome. Finally, each window was labeled according to its position: telomere, centromere, HSBs or EBRs. Using Perl scripts, we computed the density of TR and merged the positions of TR with the different types of genomic regions in the human genome.

Then, the number and the genomic position of annotated genes in the macaque genome were considered (RefSeq from the MMUL_1.0 assembly) to perform the gene distribution analysis using the BioMart browser of Ensembl (release 67). We grouped all genes with a known function in non-overlapping windows of 100 Kbp and labeled each window according to its position: telomere, centromere, HSBs or EBRs. In this case, the variable considered was gene count in each window, given that we analyzed presence/absence of genes, rather than the number of base-pairs covered by genes.

The Functional Annotation Clustering tool in DAVID (*Database for Annotation, Visualization, and Integrated Discovery*, v6.7) [50], was used in order to identify overrepresented biological terms contained in EBRs. Functional annotation clustering allows for the biological interpretation at a ‘biological module’ level of the most relevant biological terms (GO). Following algorithm’s recommendations, all clusters analyzed included a minimum of 10 genes and a maximum of 3,000 [50]. In DAVID annotation system, Fisher Exact is adopted to measure the gene-enrichment in annotation terms by means of an EASE-score, a modified Fisher Exact P-Value. EASE-scores equal or smaller than 0.05 were considered statistically significant (i.e., strongly enriched in the annotation categories). Additionally, the system uses the group Enrichment Score (a geometric mean of member’s p-values in a corresponding annotation cluster) to rank the biological significance of the genes found in a cluster. Enrichment Scores ≥ 1.5 indicated significant over-represented of gene functions.

### Biological samples

Metaphase chromosomes were prepared from peripheral blood samples obtained from one female rhesus macaque (Parc Zoològic de Barcelona, Spain). Cultures from peripheral blood samples were processed under standard conditions in order to obtain chromosome preparations as previously described [35]. Additionally, testicular tissue from an adult individual of *Cercocebus torquatus* (CTO, 2n = 42) with proven fertility was used for the study of meiotic recombination. In order to obtain spermatocyte spreads, testicular tissue was processed as previously described [64, 65].

### Immunofluorescence

Immunostaining of meiocytes was performed as previously described [64, 65]. Different sets of antibodies were used: rabbit anti-SYCP3 (Abcam), human anti-CenP (human serum CREST, a kind gift from Dr. M. Fritzel) and mouse anti-MLH1 (Pharmigen) for the detection of meiotic crossovers (COs), all of them diluted in PTBG solution (0.05% Tween 20 in PBS) 1:200, 1:200 and 1:100, respectively. Fluorochrome-conjugated secondary antibodies (all from Jackson Immunoresearch) were used for detection: goat anti-rabbit conjugated with Cy3, goat anti-human conjugated with Cy5 and goat anti-mouse conjugated with FITC diluted 1:100 in PTBG.

### Fluorescence *in situ*hybridization

BAC clones spanning evolutionary breakpoints were obtained from the human library available at CHORI (Children’s Hospital Oakland Research Institute) (Additional file 6: Table S5). DNA from BACs was extracted according to standard protocols using a commercial kit (QIAGEN Plasmid). Fluorescence *in situ* hybridization (FISH) with specific BAC clones was performed on both metaphase chromosomes and spermatocyte spreads as previously described [40, 64]. Briefly, 1 μg of the DNA plasmid was labeled with dUTP-digoxygenine by Nick Translation (Abbot kit) and ethanol precipitated with competitor DNA (Cot-1 human DNA, Invitrogen, 1 mg/ml), salmon sperm DNA (Invitrogen, 10 mg/ml) and 1/10 volume of 3 mol/L sodium acetate overnight at -20°C. The precipitated probe mix was resuspended in 14 ml hybridization buffer (50% deionized formamide, 10% dextran sulfate, 2xSSC and 0.5 mol/L phosphate), denatured 80°C for 10 min and pre-annealed at 37°C for 1 h. Preparations were visualized using a Zeiss Axioskop epifluorescence microscope equipped with the appropriate filters and a charged coupled-device camera (ProgRes® CS10plus, Jenoptik).

### Recombination analysis

The Micromeasure 3.3 software [70] was used for image analysis and for the construction of chromosome-specific recombination maps based on the relative distances between adjacent MLH1 *foci*, a marker for meiotic crossovers (COs). For each chromosome analyzed, the position of each MLH1 *foci* was recorded as a relative position (as the percentage of total length of the synaptonemal complex, SC) from the centromere, identified by the centromeric signal in each preparation as described previously [64, 65]. Using the centromere as a reference, the positions of each MLH1 *focus* were calculated along the SC, from the centromere to the telomere. Thus, for comparison among chromosomes, the position of MLH1 *foci* was expressed as the relative position of each CO to the length of the chromosome (the length of each SC was divided into 10% intervals). To convert the MLH1 *foci* to genetic distances, the number of MLH1 *foci* detected per SC was multiplied by a factor of 50 map units (1 crossover = 50 cM) [64, 65].

The effect of chromosome inversions on the CO distribution pattern was analyzed by calculating MLH1 *foci* density within inverted and non-inverted regions, considering the length (expressed in μm) for each region, so the differences due to SC lengths for each chromosome were normalized. In order to delimit the inversions directly in spermatocytes, the centromere position and the specific BAC probes labeling the breakpoint distal to centromere was used (Figure 1 and Additional file 6: Table S5). To allow for comparison among chromosomes, CO distribution was expressed as the relative position of each CO to the length of the chromosome (the length of each SC was divided into 10% intervals).

Furthermore, and in order to disentangle the centromeric effect on recombination, the CO distribution was compared between the rearranged chromosomes and the collinear one, using the last as a control. To this purpose, we simulated an inversion in the collinear chromosome, by analyzing the recombination pattern of a region of (proportionally) the same size as the observed inverted region. We constructed plots of cumulative frequency to study the pattern along the chromosome arms.

Statistical analysis was performed using JMP 10 software (SAS Institute Inc.) and IBM SPSS Statistics 20, using the Kolmogorov-Smirnov-Lilliefors test for normality, and the Kruskal-Wallis and Mann–Whitney U tests for comparisons.

## Electronic supplementary material

Additional file 1: Table S1: EBR positions involved in the macro-rearrangements (inversions spanning more than 4 Mbp, fusions and fissions) between human and macaque genomes detected in our study. (DOCX 32 KB)

Additional file 2: Table S2: EBR positions involved in the micro-rearrangements (inversions spanning less than 4 Mbp, *indels* and high-complex regions) between human and macaque genomes detected in our study. (DOCX 40 KB)

Additional file 3: Figure S1: Highly refined map of the reorganizations and evolutionary breakpoint regions in the human and rhesus macaque genomes. Representation of HSB (gray blocks) and EBRS (white regions) between human and rhesus monkey, using human as the reference genome detected by SyntenyTracker (Macaque_Synteny) and Cassis (Macaque_Cassis) algorithms, as well as the final model (Macaque_Final) (from left to right in each chromosome representation). The final model is the result of merging the outputs of both programs. Inset numbers represent the homologous rhesus monkey chromosomes. Hatched areas represent heterochromatin in the human genome. (PDF 3 MB)

Additional file 4: Table S3: Output from Functional Annotation Tool of DAVID using genes inside EBR positions (+−200 kbp) (see materials and methods for details). Annotation clusters are statistically significant when Enrichment Score is above 1.5. (XLS 43 KB)

Additional file 5: Table S4: Output from Functional Annotation Tool of DAVID using genes inside the MMU5 (see materials and methods for details). Annotation clusters are statistically significant when Enrichment Score is above 1.5. (XLS 39 KB)

Additional file 6: Table S5: Selection of human BAC clones for the EBRs detected *in silico*. (DOCX 23 KB)

## Abbreviations

HSBs: Homologous syntenic blocks
EBRs: Evolutionary breakpoint regions
SYCP3: Synaptonemal complex protein 3
SC: Synaptonemal complex
MLH1: MutL homolog 1
BAC: Bacterial artificial clones
CHORI: Children’s Hospital Oakland Research Institute
COs: Crossovers
MMU: *Macaca mulatta*
CTO: *Cercocebus torquatus*
TR: Tandem repeats
HSA: *Homo sapiens*.
