# Supplementary material for: Unraveling the effect of genomic structural changes in the rhesus macaque - implications for the adaptive role of inversions
Source: BMC Genomics. 2014 Jun 26;15(1):530. doi: 10.1186/1471-2164-15-530 (PMC4082625; doi:10.1186/1471-2164-15-530)
Supplement: Supplementary file 1 — Additional file 1: Table S1: EBR positions involved in the macro-rearrangements (inversions spanning more than 4 Mbp, fusions and fissions) between human and macaque genomes detected in our study. (DOCX 32 KB) [file 12864_2014_6198_MOESM1_ESM.docx]

**Additional file 1: Table S1:** **EBR positions involved in the macro-rearrangements (inversions spanning more than 4 Mbp, fusions and fissions) between human and macaque genomes detected in our study.** Inv, macro-inversion; OWM, Old World Monkeys; HSA, *Homo sapiens*, PTR, *Pan troglodytes*; GGO, *Gorilla gorilla*, GA-Ab, *Gorilla* ancestor; GA-A, great apes ancestor; MMU, *Macaca mulatta*. ^1^ The algorithm was unable to refine these regions owing to the sequence complexity in the pericentric region. ^2^ MMU2 centromeric position is approximately 119.7 - 122.15 kb. ^3^ Chromosome nomenclature according to Ensembl database.

| **HSA**  **chr** | | **EBR start (bp)** | **EBR end**  **(bp)** | | **Size**  **(kb)** | **Rearrangement** | **MMU**  **Chr**^3^ | **EBR start (bp)** | **EBR end**  **(bp)** | **Size**  **(kb)** | | **Ancestral**  **state** |  |
| --- | --- | --- | --- | --- | --- | --- | --- | --- | --- | --- | --- | --- | --- |
| **1** | | 120,612,240 | 145,413,095 | | 24,801^1^ | Pericentric inv. | **1** | 123,075,817 | 123,281,179 | 205.36 | | OWM |  |
|  | | 145,940,385 | 146,034,061 | | 93.67 | Pericentric inv. |  | 126,278,886 | 126,466,180 | 187.29 | |  |  |
|  | | 178,009,276 | 178,015,890 | | 6.61 | Paracentric inv. |  | 141,372,215 | 141,372,336 | 0.12 | | OWM |  |
|  | | 228,784,317 | 228,798,557 | | 14.24 | Paracentric inv. |  | 20,7626,105 | 207,730,032 | 103.93 | |  |  |
|  | | | |  | | | | | | | | |  |
| **2** | | Centromere^2^ | 114,358,060 | |  | Pericentric inv. | **13** |  |  |  | |  |  |
|  | | 132,919,179 | 133,124,106 | | 204.93 | Pericentric inv. |  | 113,708,022 | 113,869,575 | 161.55 | | HSA-PTR-GGO |  |
|  | | 138,467,646 | 138,953,450 | | 485.80 | Fission |  |  |  |  | | HSA-PTR |  |
|  | | | |  | | | | | | | | |  |
| **3** | 13,980,076 | | 14,101,468 | | 121.39 | Pericentric inv. (GA-Ab) | **2** | 60,981,144 | 61,024,942 | 43.80 | | GA-Ab |  |
|  | 15,162,938 | | 15,187,935 | | 24.99 | Pericentric inv. (GA-Ab) |  | 46,880,329 | 46,981,366 | 101.04 | | GA-A |  |
|  | 36,651,512 | | 36,654,888 | | 3.38 | Pericentric inv. (GA-A) |  | 84,782,704 | 84,834,119 | 51.42 | | GA-A |  |
|  | 75,329,050 | | 75,673,757 | | 344.71 | Pericentric inv. |  | 179,026,672 | 179,049,651 | 22.98 | | MMU |  |
|  | 126,804,402 | | 126,816,647 | | 12.25 | Pericentric inv. (GA-Ab) |  | 99,931,639 | 99,931,948 | 0.31 | | GA-Ab |  |
|  | 129,717,830 | | 129,931,627 | | 213.80 | Paracentric inv. |  | 157,455,267 | 157,455,283 | 0.02 | | MMU |  |
|  | 186,420,593 | | 186,433,796 | | 13.20 | Paracentric inv. |  | 83,813,046 | 87,632,980 | | 3,819.93 MMU | | |
|  | | | |  | | | | | | | | |  |
| **4** | | 49,064,098 | 52,709,166 | | 3,645.07^1^ | Pericentric inv. | **5** | 44,386,928 | 44,442,153 | 55.22 | | MMU |  |
|  | | 85,887,544 | 86,396,267 | | 508.72 | Pericentric inv. |  | 77,560,198 | 77,980,505 | 420.31 | |  |  |
|  | | | |  | | | | | | | | |  |
| **6** | | 108,306,519 | 108,306,831 | | 0.312 | Paracentric inv. | **4** | 103,994,154 | 103,995,302 | 1.15 | | OWM |  |
|  | | 159,350,525 | 159,353,474 | | 2.95 | Paracentric inv. |  | 155,908,076 | 155,910,246 | 2.17 | |  |  |
|  | | | |  | | | | | | | | |  |
| **7** | |  |  | |  | Fusion 7/21 | **3** | 33,125,452 | 33,129,841 | 4.39 | |  |  |
|  | | 6,872,474 | 7,040,150 | | 167.68 | Pericentric inv. |  | 45,139,061 | 45,151,951 | 12.89 | | HSA-PTR-GGO |  |
|  | | 74,959,188 | 75,039,616 | | 80.43 | Pericentric inv. |  | 140,179,552 | 140,288,113 | 108.56 | |  |  |
|  | | | |  | | | | | | | | |  |
| **9** | | 99,985,061 | 100,037,818 | | 52.76 | Pericentric inv. | **15** | 38,941,347 | 38,941,597 | 0.25 | | HSA-PTR |  |
|  | |  |  | |  |  |  |  |  |  | |  |  |
|  | | | |  | | | | | | | | |  |
| **10** | | 51,728,313 | 51,917,598 | | 189.28 | Paracentric inv. | **9** | 50,154,629 | 50,273,092 | 118.46 | | HSA-PTR |  |
|  | | 88,846,288 | 89,189,935 | | 343.65 | Paracentric inv. |  | 86,998,993 | 86,999,021 | 0.03 | |  |  |
|  | | | |  | | | | | | | | |  |
| **11** | | 3,407,845 | 3,625,505 | | 217,660 | Pericentric inv. | **14** | 3,309,277 | 3,311,247 | 1.97 | | HSA-PTR-GGO |  |
|  | | 71,531,947 | 71,626,650 | | 94,703 | Pericentric inv. |  | 69,949,028 | 69,953,608 | 4.58 | |  |  |
|  | | | |  | | | | | | | | |  |
| **17** | | 34,812,765 | 34,815,077 | | 2,312 | Paracentric inv. | **16** | 31,482,483 | 31,663,199 | 180.72 | | HSA-PTR-GGO |  |
|  | | 45,091,219 | 45,150,769 | | 59,550 | Paracentric inv. |  | 46,418,394 | 46,431,225 | 12.83 | |  |  |
|  | | 60,306,151 | 60,375,825 | | 69,674 | Paracentric inv. |  | 57,520,273 | 57,529,717 | 9.44 | |  |  |
|  | | | |  | | | | | | | | |  |
| **18** | | 15,302,248 | 18,520,346 | | 3,218.09^1^ | Pericentric inv. | **18** | 13,935,396 | 13,955,562 | 20.17 | | HSA |  |
|  | | | |  | | | | | | | | |  |
| **20** | | 26,226,969 | 29,833,279 | | 3,606.31^1^ | Pericentric inv. | **10** | 33,312,300 | 33,313,023 | 0.72 | | MMU |  |
|  | |  |  | |  | Fusion 20/22 |  | 58,957,269 | 58,983,880 | 26.61 | |  |  |
