# Supplementary material for: Unraveling the effect of genomic structural changes in the rhesus macaque - implications for the adaptive role of inversions
Source: BMC Genomics. 2014 Jun 26;15(1):530. doi: 10.1186/1471-2164-15-530 (PMC4082625; doi:10.1186/1471-2164-15-530)
Supplement: Supplementary file 2 — Additional file 2: Table S2: EBR positions involved in the micro-rearrangements (inversions spanning less than 4 Mbp, indels and high-complex regions) between human and macaque genomes detected in our study. (DOCX 40 KB) [file 12864_2014_6198_MOESM2_ESM.docx]

**Additional file 2: Table S2:** **EBR positions involved in the micro-rearrangements (inversions spanning less than 4 Mbp, indels and high-complex regions) between human and macaque genomes detected in our study.** Inv., micro-inversion; HSA, *Homo sapiens*; MMU, *Macaca mulatta*; h.c, high-complex region. ^1^Chromosome nomenclature according to Ensembl database.

| **HSA**  **chr** | **EBR start (bp)** | **EBR end**  **(bp)** | **Size**  **(kb)** | | **Rearrangement** | | **MMU**  **Chr**^1^ | **EBR start (bp)** | **EBR end**  **(bp)** | **Size**  **(kb)** |
| --- | --- | --- | --- | --- | --- | --- | --- | --- | --- | --- |
| **1** | 205,922,669 | 206,075,112 | 152.4 | | inv. | | **1** | 164,045,728 | 164,083,375 | 37.65 |
|  | 206,332,100 | 206,482,531 | 150.4 | | inv. | |  | 164,335,899 | 164,460,638 | 124.74 |
|  |  |  |  | |  | |  |  |  |  |
| **3** | 14,166,440 | 15,140,670 | 974.2 | | Indel | | **11** | 83,653,213 | 83,761,979 | 108.77 |
|  | 15,140,670 | 126,911,974 | - | | inv. (inside indel) | |  | 84,808,837 | 84,810,097 | 1.26 |
|  | 126,911,974 | 129,696,781 | 2,784.8 | | Indel | |  | 87,752,819 | 87,752,842 | 0.02 |
|  |  |  |  | |  | |  |  |  |  |
| **7** | 2,570,909 | 2,570,912 | 0.003 | | inv. | | **3** | 40,538,891 | 40,540,440 | 1.55 |
|  | 2,711,378 | 2,712,404 | 1.026 | | inv. | |  | 40,678,581 | 40,686,711 | 8.13 |
|  | 5,198,357 | 5,206,424 | 8.07 | | inv. | |  | 42,967,739 | 43,187,449 | 219.71 |
|  | 5,887,553 | 5,985,391 | 97.84 | | inv. | |  | 43,854,972 | 44,120,705 | 265.73 |
|  | 72,328,646 | 72,364,220 | 35.57 | | inv. | |  | h.c. | h.c. | - |
|  | 76,693,976 | 76,701,067 | 7.09 | | Indel | |  | h.c. | h.c. | - |
|  | 97,502,872 | 97,597,457 | 94.58 | | inv. | |  | h.c. | h.c. | - |
|  | 102,331,602 | 102,347,362 | 15.76 | | inv. | |  | h.c. | h.c. | - |
|  |  |  |  | |  | |  |  |  |  |
| **8** | 7,891,157 | 8,100,381 | 209.22 | | inv. | | **8** | 8,338,584 | 8,724,371 | 385.79 |
|  | 12,277,331 | 12,579,056 | 301.73 | | inv. | |  | 12,447,706 | 12,472,614 | 24.91 |
|  | 22,876,195 | 22,978,584 | 102.39 | | inv. | |  | 23,075,929 | 23,107,878 | 31.95 |
|  | 23,077,420 | 23,100,893 | 23.47 | | inv. | |  | 23,226,064 | 23,344,560 | 118.50 |
|  |  |  |  | |  | |  |  |  |  |
| **9** | 130,900,654 | 130,904,032 | 3.38 | | Indel | | **15** | 76,997,984 | 77,162,601 | 164.62 |
|  |  |  |  | |  | |  |  |  |  |
| **10** | 46,308,327 | 46,766,521 | 458.19 | | Indel | | **9** | h.c. | h.c. | - |
|  |  |  |  | |  | |  |  |  |  |
| **12** | 87,020,523 | 87,030,022 | 9.49 | | Indel from HSA3 | | **11** | 83,653,213 | 87,752,842 | 4,099.63 |
|  |  |  |  | |  | |  |  |  |  |
| **14** | centromere | - | - | | Fussion 14/15 | | **7** | 82,203,202 | 82,400,268 | 197.07 |
|  |  |  |  | | inv. | |  | 82,839,121 | 82,882,567 | 43.45 |
|  |  |  |  | |  | |  |  |  |  |
| **15** | 82,578,953 | 82,628,805 | 49.85 | | inv. | | **7** | 62,413,751 | 62,442,995 | 29.24 |
|  | 84,729,590 | 84,819,253 | 89.66 | | Indel | |  | 64,063,613 | 64,086,187 | 22.57 |
|  | 85,712,538 | 85,731,500 | 18.96 | | inv. | |  | 64,813,958 | 64,891,817 | 77.86 |
|  |  |  |  | |  | |  |  |  |  |
| **16** | 2,776,743 | 2,785,903 | 9.16 | | inv. | | **20** | 2,862,537 | 2,863,674 | 1.14 |
|  | 2,918,696 | 2,922,896 | 4.2 | | inv. | |  | 2,987,997 | 3,008,856 | 20.86 |
|  | 21,352,782 | 21,570,203 | 217.42 | | h.c. | |  | h.c. | h.c. | - |
|  | 21,807,865 | 21,949,594 | 141.73 | | h.c. | |  | h.c. | h.c. | - |
|  | 22,430,649 | 22,557,567 | 126.92 | | h.c. | |  | 20,995,777 | 21,069,103 | 73.33 |
|  | 28,347,569 | 28,389,580 | 42.01 | | h.c. | |  | h.c. | h.c. | - |
|  | 28,512,138 | 28,678,578 | 166.44 | | inv. | |  | 26,795,257 | 26,811,151 | 15.89 |
|  | 29,386,380 | 29,702,310 | 315.93 | | inv. | |  | 27,027,535 | 27,101,919 | 74.38 |
|  | 69,976,783 | 69,977,537 | 0.754 | | inv. | |  | 68,328,400 | 68,330,605 | 2.21 |
|  | 74,360,659 | 74,362,143 | 1.48 | | inv. | |  | 72,482,966 | 72,522,576 | 39.61 |
|  |  |  |  | |  | |  |  |  |  |
| **17** | 2,954,202 | 2,968,188 | 13.986 | | inv. | | **16** | 2,877,096 | 2,893,939 | 16.84 |
|  | 3,120,457 | 3,125,137 | 4.680 | | inv. | |  | 2,983,897 | 3,016,620 | 32.72 |
|  | 15,631,930 | 15,672,067 | 40.137 | | inv. | |  | 15,441,269 | 15,552,736 | 111.47 |
|  | 16,566,492 | 16,573,524 | 7.032 | | inv. | |  | 16,651,310 | 16,830,964 | 179.65 |
|  | 29,026,080 | 29,058,246 | 32.166 | | h.c. | |  | 26,210,707 | 26,219,799 | 9.09 |
|  | 29,332,349 | 29,333,782 | 1.433 | | h.c. | |  | 27,139,073 | 27,221,279 | 82.21 |
|  | 30,328,272 | 30,458,692 | 130.420 | | h.c. | |  | 27,471,160 | 27,476,987 | 5.83 |
|  | 43,699,652 | 43,705,984 | 6.332 | | h.c. | |  | 44,222,013 | 44,247,209 | 25.20 |
|  | 44,415,623 | 44,642,558 | 226.935 | | inv. | |  | h.c. | h.c. | - |
|  | 45,519,018 | 45,552,166 | 33.148 | | h.c. | |  | h.c. | h.c. | - |
|  | 58,052,442 | 58,205,243 | 152.801 | | inv. | |  | h.c. | h.c. | - |
|  |  |  |  | |  | |  |  |  |  |
| **18** | 10,555,774 | 10,666,059 | 110.285 | | inv. | | **18** | 2,634,444 | 2,636,247 | 1.80 |
|  | 12,034,713 | 12,046,012 | 11.299 | | inv. | |  | 3,906,554 | 3,906,561 | 0.01 |
|  |  |  | |  | |  |  | |  |  |
| **19** | 7,880,843 | 7,883,596 | 2.753 | | inv. | | **19** | 7,760,383 | 7,770,483 | 10.10 |
|  | 8,215,710 | 8,216,083 | 373 | | inv. | |  | 8,105,543 | 8,118,267 | 12.72 |
|  |  |  |  | |  | |  |  |  |  |
| **22** | 21,039,214 | 21,055,614 | 16.400 | | inv. | | **10** | 64,731,290 | 64,770,945 | 39.66 |
|  | 21,653,350 | 21,725,373 | 72.023 | | inv. | |  | 65,199,089 | 65,220,681 | 21.59 |
|  | 25,096,920 | 25,112,253 | 15.333 | | inv. | |  | 68,061,175 | 68,161,838 | 100.66 |
|  | 25,622,084 | 25,864,406 | 242.322 | | inv. | |  | 69,251,505 | 69,252,388 | 0.88 |
