# Supplementary figures and images for: Unraveling the effect of genomic structural changes in the rhesus macaque - implications for the adaptive role of inversions
Source: BMC Genomics. 2014 Jun 26;15(1):530. doi: 10.1186/1471-2164-15-530 (PMC4082625; doi:10.1186/1471-2164-15-530)

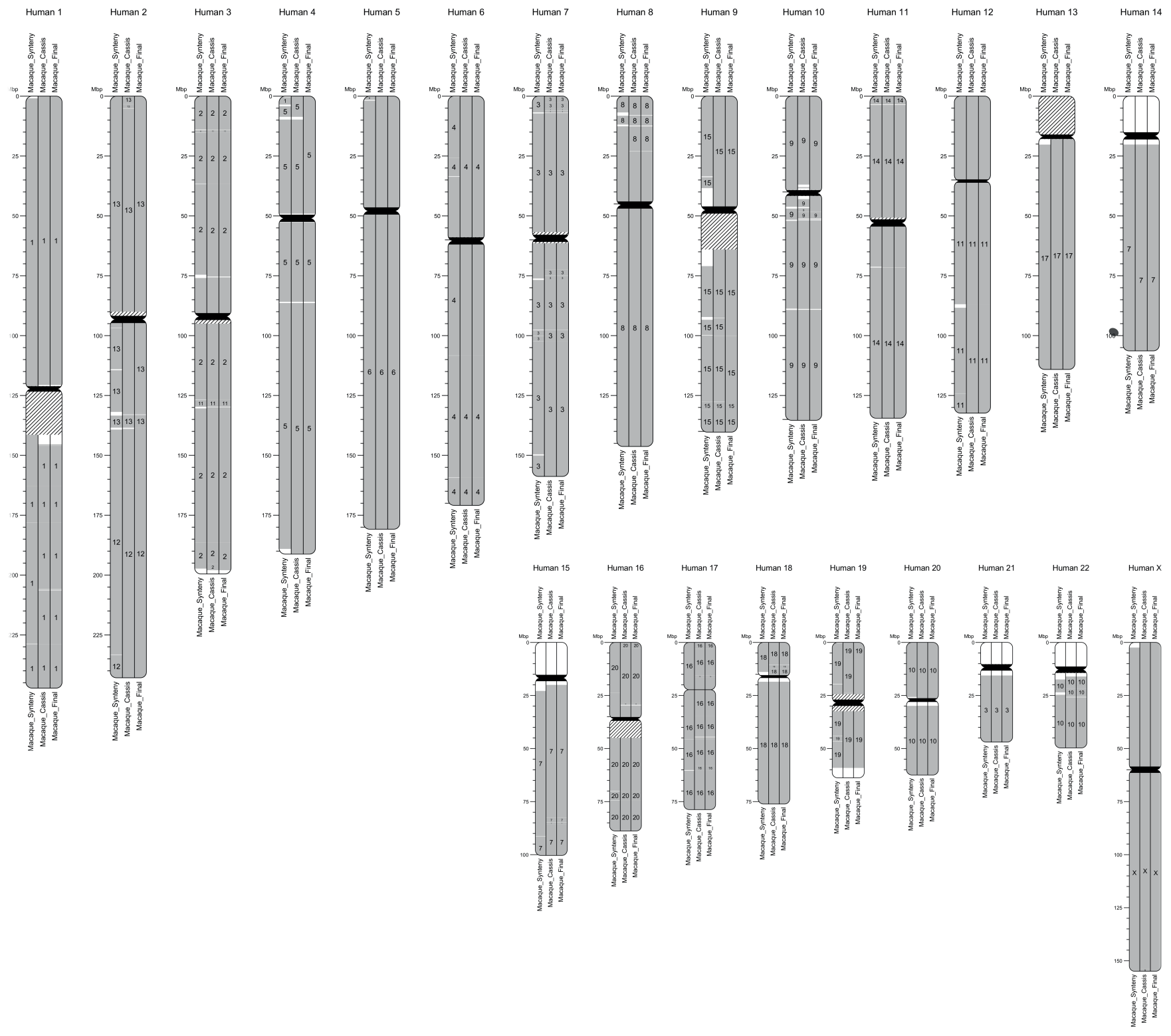

Supplement: Supplementary file 3 — Additional file 3: Figure S1: Highly refined map of the reorganizations and evolutionary breakpoint regions in the human and rhesus macaque genomes. Representation of HSB (gray blocks) and EBRS (white regions) between human and rhesus monkey, using human as the reference genome detected by SyntenyTracker (Macaque_Synteny) and Cassis (Macaque_Cassis) algorithms, as well as the final model (Macaque_Final) (from left to right in each chromosome representation). The final model is the result of merging the outputs of both programs. Inset numbers represent the homologous rhesus monkey chromosomes. Hatched areas represent heterochromatin in the human genome. (PDF 3 MB) [file 12864_2014_6198_MOESM3_ESM.pdf]
