# Supplementary material for: Unraveling the effect of genomic structural changes in the rhesus macaque - implications for the adaptive role of inversions
Source: BMC Genomics. 2014 Jun 26;15(1):530. doi: 10.1186/1471-2164-15-530 (PMC4082625; doi:10.1186/1471-2164-15-530)
Supplement: Supplementary file 6 — Additional file 6: Table S5: Selection of human BAC clones for the EBRs detected in silico. (DOCX 23 KB) [file 12864_2014_6198_MOESM6_ESM.docx]

**Additional file 6: Table S5: Selection of human BAC clones for the EBRs detected *in silico*.** *BACs that overlap with human EBR position and containing the breakpoint implicated in the rearrangement. ** BAC from a collinear chromosome used as a control of the recombination study.

| **BAC clone** | **BAC genomic position in human** | **EBR genomic position in human**  **(start-end)** | **EBR genomic position in macaque**  **(start-end)** |
| --- | --- | --- | --- |
| RP11-779N22 | Chr4: 44,815,150 – 44,834,253 | Chr4: 49,064,098 – 52,709,16 | Chr5: 44,386,928 – 44,442,153 |
| RP11-8N8* | Chr4: 85,816,998 – 86,000,969 | Chr4: 85,887,544 – 86,396,267 | Chr5: 77,560,198 – 77,980,505 |
| RP11-926D9* | Chr10: 88,901,983 – 89,087,062 | Chr10: 88,846,288 – 89,189,935 | Chr9: 86,998,993 – 86,999,021 |
| RP11-157F1** | Chr5: 95,891,189 – 96,064,042 | --- | --- |
